# Genetic and epigenetic studies of atopic dermatitis

**DOI:** 10.1186/s13223-016-0158-5

**Published:** 2016-10-19

**Authors:** Lianghua Bin, Donald Y. M. Leung

**Affiliations:** 1 The Department of Dermatology, the First Affiliated Hospital, Jinan University, Guangzhou, China; 2 Biomedical Translational Research Institute, Jinan University, Guangzhou, China; 3 Department of Pediatrics, National Jewish Health, 1400 Jackson Street, Room K926i, Denver, CO 80206 USA; 4 Guangdong Provincial Key Laboratory of Allergy & Clinical Immunology, The State Key Clinical Specialty in Allergy, The Second Affiliated Hospital of Guangzhou Medical University, Guangzhou, China

**Keywords:** Atopic dermatitis, Genetics, Epigenetic, Innate immunity, Adaptive immunity, Skin barrier, Genetic association, miRNA, DNA methylation

## Abstract

**Background:**

Atopic dermatitis (AD) is a chronic inflammatory disease caused by the complex interaction of genetic, immune and environmental factors. There have many recent discoveries involving the genetic and epigenetic studies of AD.

**Methods:**

A retrospective PubMed search was carried out from June 2009 to June 2016 using the terms “atopic dermatitis”, “association”, “eczema”, “gene”, “polymorphism”, “mutation”, “variant”, “genome wide association study”, “microarray” “gene profiling”, “RNA sequencing”, “epigenetics” and “microRNA”. A total of 132 publications in English were identified.

**Results:**

To elucidate the genetic factors for AD pathogenesis, candidate gene association studies, genome-wide association studies (GWAS) and transcriptomic profiling assays have been performed in this period. Epigenetic mechanisms for AD development, including genomic DNA modification and microRNA posttranscriptional regulation, have been explored. To date, candidate gene association studies indicate that filaggrin (*FLG*) null gene mutations are the most significant known risk factor for AD, and genes in the type 2 T helper lymphocyte (Th2) signaling pathways are the second replicated genetic risk factor for AD. GWAS studies identified 34 risk loci for AD, these loci also suggest that genes in immune responses and epidermal skin barrier functions are associated with AD. Additionally, gene profiling assays demonstrated AD is associated with decreased gene expression of epidermal differentiation complex genes and elevated Th2 and Th17 genes. Hypomethylation of *TSLP* and *FCER1G* in AD were reported; and miR-155, which target the immune suppressor *CTLA*-*4*, was found to be significantly over-expressed in infiltrating T cells in AD skin lesions.

**Conclusions:**

The results suggest that two major biologic pathways are responsible for AD etiology: skin epithelial function and innate/adaptive immune responses. The dysfunctional epidermal barrier and immune responses reciprocally affect each other, and thereby drive development of AD.

**Electronic supplementary material:**

The online version of this article (doi:10.1186/s13223-016-0158-5) contains supplementary material, which is available to authorized users.

## Background

Atopic dermatitis (AD) is the most common skin disease worldwide, affecting up to 30 % of children and 3 % of adults [1]. Together with food allergy, allergic rhinitis and atopic asthma, AD belongs to the atopic group of disorders with common characteristics of allergen sensitization, epithelial barrier abnormalities and Type 2 immune responses [2]. A number of familial studies and twin studies have demonstrated that AD is a highly heritable disease [3–9]. The rapid increase in prevalence of AD has been attributed to changes in life style and the environment. A comprehensive review by Barnes summarized genetic studies on AD before June of 2009 [10]. In her review, outcomes of 5 linkage studies and 111 targeted gene association studies were analyzed. Looking retrospectively, the year of 2009 was a time point for transition to a new era in which genome-wide association studies (GWAS) emerged as a popular approach to elucidate the genetic susceptibility of human complex diseases such as AD. Concomitantly, epigenetic alterations in response to environmental exposures opened a novel area for researchers to explore AD etiology. After June 2009, targeted gene association studies were still actively used, however, traditional genetic linkage studies for AD had been substituted by GWAS studies with large scale sample sizes in different populations. During the last 5 years, next-generation sequencing technologies have been increasingly used in AD research: two whole exome analyses were reported for the purpose of finding rare genetic variants in coding sequences of the AD genome; and high-throughput gene expression profiling assays such as RNA-sequencing technology were performed on skin biopsy specimens from AD patients. Although several reviews on the topic of AD genetics and epigenetics have been published since 2009 [11–13], more than 30 new studies have been reported since January, 2015. In this article, we summarize genetics and epigenetics studies on AD between June of 2009 and June of 2016. Studies from both adults and children with different ethnicities are included in this review. Additionally, a few studies of genome wide comparative analyses of AD versus asthma and AD versus psoriasis are discussed in the current review.

## Review

### Methods

We performed the literature search in PubMed database using the terms “atopic dermatitis” or ‘eczema” and “association”, or “gene”, or “polymorphism”, or “mutation”, or “variant”, or “genome wide association study”, or “microarray”, or “gene profiling”, or “RNA-sequencing”, or “epigenetics”, or “microRNA”, from June 2009 to present. Abstracts of the PubMed results were reviewed to identify any target gene association case–control studies, GWAS studies, gene profiling studies and epigenetics studies including both DNA methylation studies and microRNA studies of AD. Articles published in non-English languages were excluded.

This review included all results obtained from children and adults in all ethnicities. Comparative studies on “AD” and “AD plus asthma”, “AD” and “atopic march”, “AD” and “psoriasis” were included.

## Results

### Candidate gene association studies

**Fig. 1:**
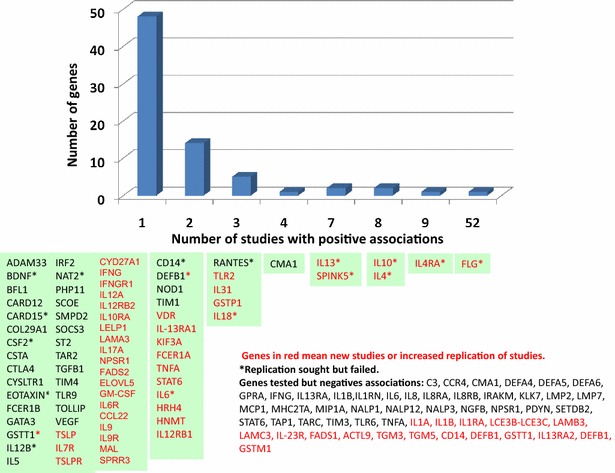
Genes associated with AD in at least 1 publication. Genes are grouped based on the reported positive association studies (see Additional file 1: Table S1 in the supplemental materials for a complete summary of 91 published studies). The *Y-axis* indicates the number of genes. The *X-axis* indicates the corresponding number of positive association reported

Candidate gene association studies have focused on the skin epidermal differentiation complex genes and Type 2 immune responses based on our understanding of the pathophysiology of AD. The review by Barnes, in 2010, had examined 81 genes that had been studied prior to June 2009 [10]. Using the same strategy of literature research as Barnes, we identified 92 published studies on candidate gene association studies in AD (Additional file 1: Table S1, Additional file 2: Reference). Among these 92 studies, 65 genes were investigated, more than half of which had at least 1 positive association. To date, *FLG* null mutation is the most replicated AD gene association. Genes in the Th2 signaling pathway are the second category that has been replicated by multiple independent studies. Beside *IL*-*4, IL*-*13, IL*-*4RA, IL13RA1, IL*-*13RA2* and *STAT6*, newly tested genes in this category are *thymic stromal lymphopoietin (TSLP),* its receptors *IL*-*7R* and *TSLPR* [14], and *IL*-*31* [15]. In the skin barrier gene category, *LAMA3* [16], *TMEM79*, *filaggrin*-*2(FLG2)* [17] and *Late Cornified Envelope*-*like Proline*-*rich 1 (LELP1)* [18] were identified to be associated with AD. The vitamin D signaling pathway is a novel pathway that has been explored in AD. In this regard, vitamin D receptor (*VDR*) polymorphisms and *CYD27A1* were found to be associated with AD severity [19–21]. Additionally, *IL10, IL6, TNFA* and *IL*-*1* family members were studied in this time frame. A few candidates from GWAS were also tested. Genes studied are summarized in Fig. 1 which include the analyses from Barnes’ review.

### Genome-wide association studies (GWAS)

Candidate gene association studies are extremely limited in scope because the selection of candidates is often from known genes with selection biases from the investigators. Thus, this approach usually does not identify novel genes or novel pathophysiological pathways. To date, of the estimated 30,000 human genes, only a very small portion of the transcriptome, have been carefully investigated. A hypothesis-free approach can significantly decrease biases and lead to identification of novel pathophysiology pathways for AD. Single nucleotide polymorphisms (SNP) are the most common class of genetic variations in humans. The haplotype structure of the human genome suggests that a set of 1 million SNP can capture approximately 90 % of genetic variation in the population. The data from the Hapmap project and development of dense genotyping chips allow GWAS assays to be effectively conducted on a large number of samples. Therefore, GWAS became a powerful method to comprehensively investigate associations between common SNP and complex diseases [22]. Using the key words “genome wide association study” and “atopic dermatitis” to search the Pubmed database, a total of 13 articles were published since 2009, 9 articles focused exclusively on AD, 4 other articles did genome-wide comparative analysis of AD with asthma/atopic march and psoriasis.

The first GWAS study of AD was published in May 2009 by Esparza-Gordillo et al. It was performed on a German cohort of 939 cases and 975 controls as well as 275 complete nuclear families with two affected siblings [23]. This study replicated *FLG* locus as an AD predisposing factor and identified a novel susceptibility region at chromosome 11q13.5 located 38 kb down-stream of *C11orf30*. Two year later in 2011, Sun et al. reported a GWAS study on Chinese Han population, *FLG* region was once more validated in Chinese population and two novel loci of 5q22.1 and 20q13.33 were identified. These two loci were validated using 1806 cases and 3256 controls from Germany [24]. Interestingly, the *TSLP* gene is located about 300 kb down-stream of the associated region of 5q22.1. In the same year of 2011, Paternoster et al. [25] published a meta-analysis of GWAS on European ancestry, in which they identified three more new risk loci for AD (11q31.1, 19p13.2, 5q31). In addition, this study reported a significant genome-wide association signal within the cytokine cluster on 5q31.1 due to two distinct signals, one centered on *RAD50/IL13* and the other on *IL4/KIF3A*. In 2012, Hirota et al. reported GWAS study findings in the Japanese population, this study added eight other novel susceptibility loci, including the major histocompatibility complex (MHC) region on chromosome 6p21 and the *IL1RL1*–*IL18R1*–*IL18RAP* locus on chromosome 2q12 [26]. In 2013, Ellinghaus et al. reported the densely genotyped results of 2425 German cases and 5449 controls using an Immunochip array [27], followed by replication in 7196 cases and 15,480 controls from Germany, Ireland, Japan, and China. Four additional novel susceptibility loci for AD were identified (4q27 *IL2/IL21*, 11p13 *PRR5L*, 16p13.13 *CLEC16A/DEXI*, 17q21.32 *ZNF652*). Adjacent genes to these loci include *TRAF6*, *RAG1*, *RAG2*, *SOCS1* and *NGFR* [27]. Additionally, Esparza-Gordillo et al. analyzed data in the public repository and validated selected markers in three different sets of cases and controls, and identified 4 SNPs, rs2040704 (*RAD50*), rs10903122 (*RUNX3*), rs2292239 (*ERBB3*) and rs2228145 (*IL6R*), as new susceptibility genes for AD. The authors further validated that the *IL6R* rs2228145 (C) genotype is associated with increased soluble IL-6R plasma levels in AD and persistent AD status using two independent population-based cohort [28].

In 2015, 3 more reports involving GWAS study and AD were published. Schaarschmidt et al. analyzed imputed SNP data from previous GWAS studies, followed by validation with additional case and control cohorts. This study validated 19 of previously established AD genetic risk loci and identified two new susceptibility loci (2q24.3 and 9p21.3) with genome-wide significance in German population [29]. Kim et al. conducted the first GWAS assay in Korean population which was aimed to identify genetic biomarkers for moderate-to-severe AD in children. Since the discovery cohort of this study only contained 246 AD cases, the results from this report are not discussed in this review [30]. To note, the AD GWAS study with the best statistic power so far is conducted by Paternoster et al. who led an international collaborated study. This study included 21,000 cases and 95,000 controls with multi-ancestry in the discovery cohort, the results were replicated in 32,059 cases and 228,628 controls, 15,539,996 variants with minor allele frequency (MAF) ≥ 1 % were analyzed [31]. This study had not only replicated 16 AD risk loci identified by previous GWAS studies, but also identified 11 more new risk loci for AD. The new loci include candidate genes of *CD207* (langerin), *PPP2R3C*, *IL*-*7R*, *STAT3* and *ZBTB10*, with known function in the regulation of innate host defenses and T cell function [32–36]. Taken together, these 8 GWAS assays and meta-analyses reported 34 AD risk loci as listed in Table 1.

**Table 1:** GWAS Assays and meta-analysis identify genetic risk genes for AD

Authors; published year, journal	Country conducted	Study objectives	Population	Method	Sample size	Reported novel loci	Reported SNP	Reported genes
Esparza-Gordillo et al. Nat Genet. 2009 May; 41(5):596–601	Germany	Identify genetic variants contributing to AD	German/European	Affymetrix Human mapping 500 K and 5.0 arrays	Discovery cohort: 939 AD, 957 Controls, 270 nuclear families Replication cohort: 1363 AD and 2739 control German; 1274 AD and 1218 European ancestry	11q13.5^ab^ 1q21.3^ab^	rs7927894(A) rs87776, rs61813875	*C11orf30* *LRRC32* *FLG*
Sun et al. Nat Genet. 2011 Jun; 43(7):690–4	China	Identify genetic variants for AD	Chinese	Illumina BeadChips	Discovery cohort: 1012 cases and 1362 controls Replication cohort: 3624 cases and 12,197 controls of Chinese Han; 1806 cases and 3256 controls of German	5q22.1 20q13.33^ab^	rs7701890 rs6010620	*TMEM232* *SLC25A46* *TNFRSF6B* *ZGPAT*
Paternoster et al. Nat Genet. 2011 Dec; 44(2):187–92	Joint effort of multiple countries in Europe, North America and Australia (EAGLE)	A collaborate effect to identify additional risk genes for AD	European descents	Affymetrix 500K, Affymetrix 5.0, Affymetrix 6.0, Illumina 317 K/610 K, Illumina 610 quad, Meta analyses	Discovery cohort: 5606 cases and 20,565 controls from 16 populations Replication cohort: 5419 cases and 19,833 controls from 14 studies	11q13.1^a^ 19p13.2^a^ 5q31^a^	rs479844 rs2164983 rs2918307 rs2897442	*OVOL1* *ACTL9/ADAMTS10* *KIF3A* *IL*-*13*-*RAD50*
Hirota et al. Nat Genet. 2012 Nov; 44(11):1222–6	Japan	Identify genetic variants for AD	Japanese	Illumina Human OmniExpress Bead Chips	Discovery cohort: 1472 cases and 7971 controls Replication cohort: 1856 cases and 7021 controls	2q12.1^ab^ 6p21.32^ab^ 11p15.4^a^ 3p21.33 3q13.2^ab^ 7p22.2^b^ 10q21.2^ab^ 20q13.2^b^	rs13015714 rs176095 rs878860 rs6780220 rs12634229 rs4722404 rs10995251 rs16999165	*IL1RL1*-*IL18R1*-*IL*-*18RAP* *GPSM3(MHC region)* *OR10A3/NLRP10* *GLB1* *CCDC80* *CARD11* *ZNF365* *CYP24A1*-*PFDN4*
Ellinghaus et al. Nat Genet. 2013 Jul; 45(7):808–12	Germany	To better define risk variants and identify additional susceptibility loci for AD	German Irishman Chinese Japanese	ImmunoChip array Meta-analysis	Discovery cohort: 2425 cases and 5449 controls of German Replication cohort: 7196 cases and 15,480 controls from Ireland, Japan and China	2q12.1 4q27^ab^ 11p13^ab^ 16p13.13^ab^ 17q21.32^b^	rs759382 rs17389644 rs12295535 rs2041733 rs16948048	*SLC9A4* *IL2/IL21* *PRR5L* *CLEC16A*-*DEX* *ZNF652*
Esparza-Gordillo et al. J Allergy Clin Immunol. 2013; 132(2):371–7	Germany	To identify novel genetic risk factors for AD	European	Affymetrix 5.0 or Illumina HumanHap 300	Discovery cohort: 2895 cases and 2448 subjects 1st replication cohort: 1429 cases and 1737 controls 2nd replication cohort: 2806 cases and 5068 controls	1q21.3^a^	rs2228145	*IL6R*
Schaarschmidt et al. J Allergy Clin Immunol. 2015; 136(3):802–6	Germany	To identify novel genetic risk factors for AD	German	Affymetrix Genome-wide human SNP Array 6.0(1000 k).	Discovery cohort: 870 cases and 5293 controls Replication cohort: 1383 cases and 1728 controls	2q24.3 9p21.3	rs6720763 rs10738626	*XIRP2(CYMA3)* *DMRTA1*
Paternoster et al. Nat Genet. 2015 Dec; 47(12):1449–56	Joint effort of multiple countries cross continents	A collaborate effect to identify additional risk genes for AD cross different ethnicity	European Asian African Latino	Meta-analysis	Discovery cohort:21,399 cases and 95,464 controls Replication cohort: 32,059 cases and 228,628 controls	14q13.2 11q24.3 1q21.2 8q21.3 10p15.1 5p13.2 2p25.1 2p16.1 17q21.2 3p21.1 2p13.3	rs2038255 rs7127307 rs7512552 rs6473227 rs6602364 rs10214237 rs10199605 rs4643526 rs12951971 rs7625909 rs112111458	*PPP2R3C* -*/EST1* *C1orf51/MRPS21* *MIR5708/ZBTB10* *IL15RA/IL2RA* *IL7R/CASPL* *LINC00299* *PUS10* *STAT3* *SFMBT1/RFT1* *CD207/VAX2*

^a^Previous loci replicated in Paternoster et al. 2015 multi-ancestry genome-wide association study

^b^Previous loci replicated in Schaarschmidt et al. 2015 GWAS analysis focusing on German population

Children with AD often develop asthma and other allergic conditions later in life. This phenomenon is termed “the atopic march” [37]. Identification of genetic risk factors for the atopic march is important for the development of asthma prevention strategies. Weidinger et al. conducted a GWAS study to specifically examine the genetic differences between the AD endophenotypes “AD plus asthma” and “AD no asthma” [38]. In this study, 1563 cases of children-onset AD with known asthma status and 4054 European controls were genotyped as discovery cohort. Association variants were further assessed for a replication cohort including 2286 European cases and 3160 European controls. The results found that *FLG* locus, 5q31 between *RAD 50* and *IL*-*13* locus, 6p21 MHC locus and 11q13.5 locus were associated with the co-morbidity of AD and asthma. Using more stringent criteria of inclusion for the atopic march cases (eczema up to the age of 3 years and asthma up to age of 16 years) as compared to the study of Weidinger et al., Marenholz et al. conducted a meta-analysis to search for genetic markers of the atopic march in European descents [39]. This study validated the results of the Weidinger et al. report. Additionally, two novel loci (6p12.3/*EFHC1* and 12q21.3/*SLC6A15*) for the atopic march were reported. Importantly, both studies demonstrated a strong contribution of AD risk genes to subsequent asthma occurrence in the atopic march, supporting the epidemiology observation that infantile eczema is a predisposing factor for asthma (OR 4.33; 95 % confidence interval 3.72–5.01, p < 0.0001) [39].

The differences between AD and psoriasis (another common cutaneous inflammatory disease) were also investigated using meta-analysis of GWAS data [38, 40, 41]. AD is associated with Type 2—polarized immune responses, allergen sensitization and recurrent microbial skin infections, while psoriasis is associated with Type 1–polarized immune responses and is not associated with skin infections. Corresponding to their clinical phenotypes, opposing genetic effects were seen on Th2 locus and loci related to Th1 cytokines and host anti-viral genes between AD and psoriasis. Some genetic risk loci are in concordance for AD and psoriasis, suggesting that these two common dermatological disease share some genetic and inflammatory features.

### Gene expression profiling assays

Disease related genetic variants usually either alter gene expression or change the function of gene products by changing protein amino acid structure. Epigenetic modification and microRNA are important mechanisms that can also alter gene expression. Investigation of the transcriptome in disease relevant tissues and cells is therefore an ideal strategy to identify molecular signatures of complex diseases. Guttman-Yassky et al. performed high-throughput expression profiling on skin biopsies from AD lesions compared with health control subjects [42]. This study observed that the expression of a large number of keratinocyte terminal differentiation genes were reduced in AD as compared to normal subjects. The affected genes included *filaggrin, loricrin (LOR)*, *involucrin*, late cornified envelope protein *LCE2B*, S100 fusion gene *TCHH* and multiple S100 family members, etc. This study indicated AD was associated with broad defects of epidermal cornification in AD skin lesion. These results validated a previous microarray profiling study by Sugiura et al. which revealed down-regulation of LOR and FLG in AD skin lesions [43]. More recently, the Guttman-Yassky group used RNA-sequencing technology to compare the transcriptomes of nonlesional and lesional skin from patients with moderate-to-severe AD. This study identified increased expression of a novel *TREM*-*1* signal pathway as well as *IL*-*36* in AD [44]. Using laser capture microdissection to separate epidermis and dermis of AD lesional and nonlesional skin compared with the expression profiles of normal skin transcriptomes, Esaki et al. once again demonstrated that AD lesions had down-regulation of genes encoding skin barrier proteins including *FLG*, *LOR*, *CLDN4* and *CLDN8*; and elevated gene expression of Th2 and Th17 cytokines such *CCL22*, *CCL26*, *TSLP* and *IL*-*22* etc. [45].

Loss-of-function mutations of the gene encoding FLG are the most significant genetic risk factor for AD. Cole et al. conducted a transcriptome profiling study using a RNA-sequencing approach to compare non-lesional skin from AD with site-matched samples from healthy controls [46]. This study found that differentially expressed genes between normal and AD subjects were enriched in pathways involved in the extracellular space, lipid metabolism and stress response. When the whole transcriptome data-set was stratified according to FLG genotype, FLG deficient skin expressed a type-I interferon-mediated stress response.

A small subset of AD are susceptible to disseminated herpes simplex virus skin infections, also called “eczema herpeticum” (EH). Our lab recently compared the transcriptomic changes of peripheral blood mononuclear cells (PBMCs) between AD with a history of EH (ADEH+) and AD without a history of AD (ADEH−). The results demonstrated that unstimulated ADEH+ and ADEH–PBMC had similar transcriptomes. However, following stimulation with herpes simplex virus 1, PBMCs from ADEH+ had distinct transcriptome profiles as compared to ADEH−, with striking down-regulation of anti-viral cytokines including both type I and type III interferons. These results are indicative of defective innate immune responses in ADEH+ subjects [47].

### Epigenetic studies of AD: DNA modification and microRNAs (miRNAs)

The prevalence of AD has been increasing too rapidly to be accounted for by shifts in genetic variation. These changes are thought to be due to environmental factors such as industrialization or the western life style associated with increased stress, more sedentary life style, obesity, low vitamin D level, overuse of antibiotics, etc. [48]. Birth cohort studies suggest that both indoor and outdoor pollution are risk factors for the development of AD [49]. There is increasing evidence showing that environmental factors regulate gene expression through genomic DNA modifications and miRNA mechanisms [50]. A study by Liu et al. suggested that diesel exhaust resulted in hypermethylation of CpG sites in the *IFNG* gene promoter and hypomethylation of the *IL4* gene promoter. This was positively correlated with increased induction of IgE in response to intranasal challenge with the allergen, Aspergillus fumigatus [51]. This observation is further supported by a recent study that poly-aromatic hydrocarbon such as benzopyrene, derived largely from incomplete combustion of organic material, such as fossil fuels, coal, wood and tobacco, is associated with increased serum IL-4 in children with asthma [52]. Several studies demonstrated that Benzo[a]pyrene can decrease global DNA methylation by inhibition of DNA methytransferase expression and interfere with assembling of the methylation machinery [53–55]. Another study showed that children with prenatal tobacco exposure had a global hypomethylation at AluYb8 repeat element in buccal cells collected from mouth swab [56].

Several epigenetic studies targeting AD have been conducted and summarized in Table 2. A Chinese group led by Lu et al. have reported that hypomethylation of the promoters of *TSLP* and *FCER1G* are responsible for gene over-expression in AD [57, 58]. A German group recently reported an integrated epigenetic and transcriptomic analyses using epidermal lesions from AD patients in comparison with healthy control epidermis. The results showed that methylation status in AD skin lesions was strikingly different from healthy control epidermis and was partly correlated with the transcript levels of genes involved in epidermal differentiation and immune response [59]. In addition, two preliminary studies on *FLG* gene methylation have been reported but gave conflicting results [60, 61].

**Table 2:** Epigenetic Studies of AD

Cell/tissue types	Epigenetic assay	Significant findings	Reference
Monocytes	FCER1G methylation	Demethylation of FCER1G promoter leading to its overexpression in AD.	[57]
Skin biopsies	TSLP methylation	Demethylation of TSLP promoter resulting in its over-expression in AD lesion.	[58]
Skin biopsies	DNA methylation profiling	DNA methylation profiles are striking different in AD skin lesions as compared to healthy controls.	[59]
Buccal cells	FLG methylation	Methylation of the FLG promoter does not affect gene expression and allergy.	[60]
Whole blood	DNA methylation profiling	Methylation of one site cg07548383 in FLG is associated with increased AD risk.	[61]
Skin biopsies	miRNA profiling	Up-regulation of 10 miRNA and down-regulation of 34 miRNAs in AD skin lesions as compared to healthy control skins. miR-155 overexpression was validated in T cells within skin lesion. *Staphylococcal* superantigens induce miR-155, it targets CTLA-4.	[63]
Serum and urine	miRNA profiling	miR-203 and miR-485-5p were significantly up-regulated in serum of AD	[65]

Aside from modification of genomic DNA to transcriptionally regulate gene expression, miRNA mediated post-transcriptional regulation is another type of epigenetic gene expression regulation. miRNAs are a class of non-coding molecules that bind to the 3′-UTR of target mRNAs and regulate translation [62]. They are highly efficient in fine-tuning gene expression, exerting subtle yet significant effects throughout the genome, and their expression can be induced by environmental factors such as microbes and toxins. A few studies have explored whether miRNA are involved in AD pathogenesis. The first report by Sonkoly et al. used PCR-based miRNA arrays to compare healthy and AD lesional skin, and identified 44 miRNAs that were significantly different between AD and healthy controls with 34 down-regulated and 10 up-regulated miRNAs. The authors further validated miR-155 as significantly over-expressed in infiltrating T cells in AD skin lesions [63]. The authors found that environmental factors such as dust mite allergen and staphylococcal superantigens could induce miR-155 expression in atopic skin and identified the immune suppressor, *CTLA*-*4*, as its target gene [63]. Recently, a different group reported that miR-155 could be induced by LPS and IL-10 was its target gene [64]. In addition, a study conducted in a northern Chinese cohort found that miR-203 and miR-483-5p were significantly upregulated in serum of children with AD as compared with healthy children. The level of miR-483-5p in serum was significantly associated with AD and other atopic conditions including rhinitis and/or asthma [65].

## Discussion

### Limitations of current genetic and epigenetic studies on AD

Currently, all published studies of gene profiling assays with AD have involved relatively small sample sizes. Thus, replication and validation are significantly needed. Comparing the three different approaches, GWAS assays provide a relative high degree of replication, suggesting that the discovered genetic risk loci are robust. One caveat of the existing GWAS studies is that the SNPs identified in these studies often have a minor allele frequency greater than 1 %; therefore GWAS results cannot fully explain AD heritability due to their limited power to detect common variants with only a small effect [29]. The “missing heritability” of AD may require identification of rare genetic variants using deep sequencing technology such as the whole exome sequencing and whole genome sequencing. Two exome analyses of AD were reported: the study by Suzuki et al. did exome-sequencing on 37 AD with the extreme phenotype of serum IgE > 1000 units. The positive hits from this discovery cohort were then validated in replication cohort including 469 AD and 935 controls. This approach identified a rare genetic variant rs199691576(A/G) in *CYP27A1* that is associated with AD of high serum total IgE [21]. Another study was performed on 60 Africa American patients with AD and found *filaggrin*-*2* variation is associated with a more persistent phenotype [17]. The Atopic Dermatitis Research Network (ADRN) funded by National Institute of Health/National Institute of Allergy and Infectious Diseases have initiated whole genome sequencing analyses on AD genomes and the results are currently being analyzed [66].

Epigenetic studies of AD are currently at the stage of exploratory with small sample sizes investigated. Since the epigenetic modifications are tissue-specific and often result in gene expression changes, it is the best to investigate epigenetic alteration and gene profiling on the same tissue or cells taken from human subjects simultaneously. Currently, only one study did so [59].

Based on results of target gene association assays, GWAS and transcriptome profiling analyses as well as epigenetic studies for AD accomplished so far, genes involved in disease pathogenesis mainly fall into two pathophysiologic groups: skin barrier genes and immune response genes. In most patients, both of these two major pathways cross talk with each other to form complex pathways leading to the development of AD.

### Filaggrin and other skin barrier genes

It is now commonly accepted that skin barrier dysfunction is an essential feature for the pathogenesis of AD [67]. A disrupted skin barrier allows penetration of microbes, allergens, toxins and pollutants, leading to skin inflammation, allergen sensitization and bacterial colonization. Normal epidermal skin barrier function requires an intact stratum corneum and tight junctions in the stratum granulosum. An earlier linkage study for AD had implicated chromosome 1q21 where a very large cluster of genes involved in the epidermal differentiation process is located. This group of genes is also referred to as “the epidermal differentiation complex (EDC)” and includes *FLG*, *loricrin*, *involucrin*, *small proline*-*rich proteins (SPRRs)*, S100A family, S100-fusion protein family and late cornified envelope proteins. To date, *FLG* from the EDC cluster is the most significant risk factor for AD pathogenesis, and two null mutations R501X and 2282del4 of *FLG* in Caucasians have demonstrated the strongest association for AD (18 and 48 % of moderate to severe AD, respectively) [68]. Furthermore, the frequency of R501X in AD with a history of EH is three times higher (24 vs 8 %, respectively, OR 11.8 vs 6.2; P = 0.0008) [69] than ADEH− subjects. The association of *FLG* null mutation with AD and EH have demonstrated ethnic differences: In Asians, *FLG* P478 S and C3321delA are associated with increased risk to AD, the atopic march and recurrent skin infection [70–76]; In African populations, *FLG* mutations are not common [77–80]. In contrast to European and Asian AD, loss-of–function mutations in *FLG2*, but not *FLG*, are associated with increased risk in African American children [17].

The *FLG* gene comprises three exons. The third exon is the largest and consists of nearly identical tandem repeats of about 972 base pair in length and has allelic variants of 10, 11 and 12 repeats [81]. A study of an Irish case–control cohort found that the number of repeats was significantly lower in AD cases than controls, suggesting that common copy number variations contributes to AD risk [82].

Because AD is a complex disease caused by the combination of genetic variation and environmental factors, it is important to evaluate the impact of gene-environment interactions. There are two recent articles reported interesting results on the interaction of environmental factors and *FLG* gene mutations [83, 84]. Phthalates are chemicals commonly used in a variety of cosmetic and personal care products. Wang et al. recently reported that phthalate metabolite levels are significantly associated with increased AD risk in Chinese children, and children with *FLG* P478S mutations have increased skin absorption of phthalate. These data suggest that *FLG* mutations may increase skin permeability leading to higher skin absorption of phthalate and thus confer an increased risk for AD [84, 85]. Another recent study conducted in Europe reported that maternal *FLG* mutations increase the risk of AD in children and this increased risk is independent of mutation inheritance, indicating that maternal *FLG* mutations can act as strong environmental risk factors for the offspring [83].

There is evidence that additional EDC genes may be associated with AD [86]. Recently, a case–control study found that a 24-bp deletion in *SPRR3* was associated with AD in European cohorts [87]. However, deletion of *LCE3B* and *LCE3C* genes is not associated with AD in Caucasians [88]. Although a case–control study evaluating polymorphisms across 21 EDC genes in a German cohort did not find evidence for associations apart from *FLG*, several transcriptomic profiling studies have reported that EDC genes of IVL, LOR and LCE2B, and the cell–cell adhesion protein, CDSN, were significantly down-regulated in AD skin lesions, suggesting that elevated inflammatory cytokines in the disease loci also play an important role for dysregulation of epidermal barrier genes [42, 43].

Tight junctions in the granular layer of the epidermis play an important role in maintaining skin barrier integrity for regulation of transepidermal water loss. The claudin family represents one type of tight junction transmembrane proteins [89, 90]. The gene expression of claudin-1 (CLDN1) has been found to be reduced in the AD skin. Both Th2 cytokines and genetic variants were responsible for CLDN1 reduction in AD [91, 92]. Desmosomes are also important structures to maintain skin barrier functions that connect the cell surface to the intermediate filament cytoskeleton [93]. Samuelov et al. reported that loss-of-function mutations in gene *DSG1*, which encodes an important desmosome protein desmoglein 1, cause severe dermatitis, multiple allergies and metabolic wasting in human [94]. Additionally, two groups demonstrated that homozygous mutation of *TMEM79* is responsible for the spontaneous dermatitis phenotype in flaky tail mice which was originally thought caused solely by *FLG* mutations [95–97]. Sasaki et al. demonstrated that TMEM79 has a function in the lamellar granule secretory system [95], indicating that this skin barrier deficiency can lead to AD skin inflammation. Moreover, a missense SNP (rs6694514) of human *TMEM79* was identified to significantly associate with AD using a meta-analysis of 4245 AD cases and 10,558 population-matched control subjects [96]. Several other genes including *LAMA3* encoding the alpha chain of laminin 5, *OVOL1* and *ACTL9* have been reported to be associated with AD [16, 25]. *ACTL9* variants, however, were not replicated in two independent studies [98, 99].

The *serine protease inhibitor of Kazal type 5* (*SPINK5*) gene encoding lympho-epithelial kazal type-related inhibitor type 5 (LEKTI) is a crucial protease inhibitor for epidermis homeostasis. This gene was found to have loss-of-function mutations in Netherton syndrome, a severe autosomal recessive skin disease including AD and sensitization [100]. Gene variants of *SPINK5* was found to associate with AD in a Japanese population [101]. Functional studies demonstrated that enhanced protease activity due to defective function of SPINK5 led to increased proteolytic activity within the epidermis, subsequently resulting in reduced DSG1 and *FLG*, as well as enhanced TSLP expression. These changes all contribute to AD pathogenesis [102, 103]. Additionally, there is evidence shown that deficient FLG in keratinocytes leads to increased IL-1 and TSLP expression [104, 105]. These studies demonstrate that functional lack of epidermal enzyme inhibitors and structural proteins not only compromise the skin barrier integrity, but also mediate immunologic responses of allergic inflammation.

### Adaptive/innate immune response genes

Since AD is associated with allergen sensitization, elevated serum IgE and increased expression of Type 2 cytokines (IL-4, IL5, and IL-13) in both unaffected skin and skin lesions of AD, candidate gene studies for AD have also focused on the Th2 pathway. Indeed, the GWAS assays have repeatedly identified AD genetic risk loci around Th2 genes regions at 5q31. Genetic variants of genes in the Th2 signaling pathway including *IL4*, *IL13*, and the *IL4* receptor are positively associated with AD [10, 106–112]. *IL*-*4* receptor down-stream genes such as *STAT6* have also been reported to have positive correlations to AD as well [113–115]. Additionally, gene variants in the alpha and beta chains of the high-affinity IgE receptor (*FCER1A/B*) have been implicated in AD pathogenesis [116].

The Th2 cytokine, IL-31, is also increased in AD lesions and serum [117–121]. It is not only involved in AD inflammatory responses and dysregulation of skin barrier [122–124], but also causes severe itching [125–127]. Recently, antibodies against IL-31 receptor A has begun to be tested in clinical trials to treat AD and reduced itching [128]. *IL31* polymorphisms have been reported in AD by several groups [15, 129, 130].

Both GWAS studies and targeted gene association studies have provided evidence for the association of AD risk with *TSLP* gene variants [14, 24]. TSLP can be induced in epidermal epithelial cells by a variety of stimuli including scratching, viral infections, inflammatory cytokines, protease allergens, bacteria and bacterial products [131]. The expression of TSLP is significantly increased in AD skin lesions [45]. The major function of TSLP is to promote Th2 immune response, thus it has been considered to play an important role in AD pathogenesis. A targeted gene association study reported that genetic variants in *TSLP* are associated with AD, and the association is stronger in patients with the ADEH+ phenotype [14]. Additionally, polymorphisms of *IL*-*7R* (T244I at exon 6 and T46I at exon 2), a TSLP receptor, are associated with AD [132]. Two other epithelium-derived Th2-promoting cytokines IL-33 and IL-25 also play an important role in the pathophysiology of AD [133, 134]. No genetic polymorphism, however, of these two genes has been reported to be associated with AD.

The elevated Type 2 response in AD could be the secondary effect of impaired Th1 responses or reduced inhibitory feedback. Based on this notion, targeted gene association studies have also been performed on genes in Th1 responses and immune suppression genes. IL12B, IL12 receptor beta 1(IL12RB1) and IL-18 promote Th1 development [135–137]. There are studies reported that both *IL12B* A1188C and *IL12RB1* A-111T were associated with the risk of AD in a Japanese population [138, 139], and *IL18* variants were associated with AD in both German and Korean populations [140, 141]. Both suppressor of cytokine signaling 3 (SOCS3) and IL-10 can suppress adaptive immune responses [142, 143]. *SOCS3* polymorphism was associated with elevated expression in European AD patients [144]; and *IL10* (-819 and -592 promoter polymorphisms) was found to be associated with AD in a Korean population [145].

Aside from AD patients prone to EH, AD is often complicated by recurrent bacterial infection and other types of virus infection. The most common bacterial pathogen for AD is *Staphylococcus aureus*. Molluscum contagiosum virus can cause eczema molluscatum, and exposure to vaccinia virus can cause eczema vaccinatum after smallpox vaccine inoculation [146]. The increased prevalence of skin infections in AD suggest that the innate defense system is impaired in AD skin. Indeed, genetic variants in multiple genes functioning in pattern recognition receptors (PRR) have been implicated in the pathogenesis of AD. A number of genes in PRR signaling pathways, including *TLR2*, *TLR9*, *CD14*, *TOLLIP*, *MYD88*, *MAL*, *NOD1*, *NOD2* and *NALP12*, has been reported to be associated with AD [147–149]. Polymorphisms in anti-microbial peptides of S100 protein, human defensins α and β and sphingosine have also been implicated in AD pathogenesis [147, 148]. In addition to the direct effect of genetic modifications in innate immune response genes, the attenuation of the normal antimicrobial response caused by overexpression of Th2 cytokines in skin is especially relevant in AD. For example, it has been demonstrated that Th2 cytokines can inhibit gene expression of human β-defensin 3 and LL-37 in epidermal keratinocytes [150, 151]. Taken together, both genetic variation/mutation and acquired impairment of innate immune responses may contribute to Th2 polarization of AD. However, the genetic loci identified by GWAS need further fine mapping in order to identify the genes exactly involved; most of the associations mentioned by candidate gene assays here involved relatively small cohorts, and replication in independent large populations are needed. It is critical to note that functional validation for these candidate genes represents a significant unmet need in the field, and that should be part of the future direction in AD research.

## Conclusions

**Fig. 2:**
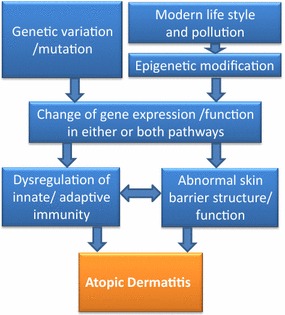
The schematic illustration of AD etiology. Genetic and epigenetic reasons lead to the alteration of gene expression and function of AD associated genes. AD associated genes majorly belong to two pathways: skin barrier and innate/adaptive immunity. Dysregulation of innate/adaptive immune responses and impaired skin barrier reciprocally affect each other to drive AD development

In summary, candidate genes for AD suggest that epidermal barrier dysfunction, enhanced Th2 immune signaling, weakened innate immune responses, IL-1 signaling and the vitamin D pathway etc. have roles in the pathogenesis of AD. Epigenetic studies also indicate the modifications of genes involved in these pathways as well. The dysfunctional epidermal barrier and immune responses reciprocally affect each other, and thereby drive the development of AD (Fig. 2). Interventions targeting either of these pathways can lead to remission of this disease.

In the future, it will be important to identify biomarkers with prognostic and predictive value for AD. Such biomarkers will lead to opportunities for precision medicine in AD. Nevertheless, research accomplishment to date further confirmed that *FLG* mutations and the Type 2 pathway are major risk factors for AD. Therapeutic reagents improving FLG function and biologics blocking Th2 cytokines such as anti-IL-4 receptor alpha, anti-IL-4/IL-13 or TSLP [152, 153] are evolving as treatment for severe AD patients. Based on GWAS studies of the atopic march, arrest of AD in infancy may be beneficial for prevention of asthma in this subset of AD patients.

## Additional files

**Additional file 1: Table S1.** Candidate gene association studies of AD from June 2009 to June 2016.

**Additional file 2: References.** References of candidate gene association studies in Table S1.

## Abbreviations

AD: atopic dermatitis
ADEH+: atopic dermatitis with a history of eczema herpeticum
ADEH-: atopic dermatitis without a history of eczema herpeticum
EDC: epidermal differentiation complex
FLG: filaggrin
GWAS: genome-wide association study
PRR: pattern recognition receptor
SNP: single nucleotide polymorphism
Th: T helper lymphocyte
TLR: toll-like receptor
TSLP: thymic stromal lymphopoietin
