# Supplementary material for: Genetic and epigenetic studies of atopic dermatitis
Source: Allergy Asthma Clin Immunol. 2016 Oct 19;12:52. doi: 10.1186/s13223-016-0158-5 (PMC5069938; doi:10.1186/s13223-016-0158-5)
Supplement: Supplementary file 1 — Additional file 1: Table S1. Candidate gene association studies of AD from June 2009 to June 2016. [file 13223_2016_158_MOESM1_ESM.docx]

**TABLE E1.** Genes associated with atopic dermatitis in at least one study

| **Gene**  **(alias)** | **Chromosome** | **Variants** | **Association** | **Population/country** | **No of subjects (cases/controls)** | **Reference** |
| --- | --- | --- | --- | --- | --- | --- |
| *ACTL9* | 19p13.2 | rs2164983 | No, AE | Italian | 359/778 | E43 |
| *ACTL9* | 19p13.2 | rs2164983 | No | Chinese | 235/200 | E89 |
| *CCL22* |  | rs4359426 | Yes | Japanese | 916/1032; 1034/1004 | E54 |
| *CD14* | 5q31.1 | rs2569190 | No | Korean | 412 infants | E26 |
| *CYP27A1* | 2q35 | rs199691576 | Yes | Japanese | 37 AD;  469/935 | E1 |
| *DEFB1* |  | -20 G/A (rs11362), -44 C/G (rs1800972), -52 G/A (rs1799946） | No | northeast Brazil | 96/191 | E71 |
| *ELOVL5* |  | Gene expression | Yes, AE | Spain | 20/104 4-Year-Old Children | E31 |
| *FADS1* |  | Gene expression | No, AE | Spain | 20/104 4-Year-Old Children | E31 |
| *FADS2* |  | Gene expression | Yes, AE | Spain | 20/104 4-Year-Old Children | E31 |
| *FCER1A* |  | rs2427837 A/G | Yes | Chinese, Han | 97 AD/491 | E52 |
| *FCER1A* |  | rs2251746 | Yes with AD and increase IgE levels | Korean | 175/56 | E58 |
| *FLG* | 1q21 | R501X/2284del4 | Yes | Polish | 152/123 | E6 |
| *FLG* | 1q21 | R501X/2284del4 | Yes with increased IgE levels | Polish | 105 AD | E7 |
| *FLG* | 1q21 | 2284del4 | Yes | Polish | 152/104 | E8 |
| *FLG* | 1q21 | not specify which SNP | Yes | Denmark | 1547 children birth cohort | E9 |
| *FLG* | 1q21 | C2284del4/R501X/R2447X/S3247X | Yes | Germany, UK, Sweden | two samples including 759 and 450 AD families | E13 |
| *FLG* | 1q21 | R501X, 2282del4, R2447X | FLG mutation and AD sensitization to food | Danes | 2308 | E14 |
| *FLG* | 1q21 | P478S | FLGP478S is associated with increased chance to develop asthma | Taiwanese | 397 AD | E17 |
| *FLG* | 1q21 | P478S | Yes | Taipei | 106/347 children | E18 |
| *FLG* | 1q21 | 2282del4, R501X, R2447X, 3702delG, S3247X, and the 12-repeat allele (rs12730241) | Yes on 2282del4 | Western siberian | 460 children | E19 |
| *FLG* | 1q21 | R501X, 2282del4, R2447X, S3247X | No, FLG mutation | Ljubljana | 241/164 | E21 |
| *FLG* | 1q21 | rs3126065, rs2786680, rs1933063, rs3814300, rs2485518, rs3814299 | No, FLG mutation | Caucasian | 106/105 | E22 |
| *FLG* | 1q21 | p.S2554*, p.S2889*, p.S3296* | No, FLG mutation | Japanese, Ishigaki Island | 127/594 children | E24 |
| *FLG* | 1q21 | c.3321delA | Yes | Chinese, Han | 1080/908 | E25 |
| *FLG* | 1q21 | P478S | Yes | Korean | 322/326 | E28 |
| *FLG* | 1q21 | E2422X, R501X, 3321delA | Yes on 3321delA | Korean | 1430/862 | E35 |
| *FLG* | 1q21 | R501X or 2282del4 | Yes on self-reported food allergy and alcohol sensitivity, but not with OAS | Copenhagen | 3471 | E38 |
| *FLG* | 1q21 | 3222del4, 3321delA, K4671X | No | Chinese, Han | A group of 100 family trios (a total of 300 members) | E46 |
| *FLG* | 1q21 | 3321delA, K4671X | No | Chinese, Han | 249 AD | E48 |
| *FLG* | 1q21 | FLG null mutation | Yes with increased recurrent infection | SingaporeanChinese | 228 AD | E57 |
| *FLG* | 1q21 | full sequence FLG to find mutation and association ananlysis | Yes，filaggrin null mutations | Singaporean Chinese and European | 425/440 | E62 |
| *FLG* | 1q21 | R501X, 2282del4 | Yes | Eastern European/Polish descent | 163/204 | E63 |
| *FLG* | 1q21 | full sequence FLG to find mutation and association ananlysis | No | Italian | 5791/26454 | E66 |
| *FLG* | 1q21 | P478S | Yes | Chinese | 116/212 children | E67 |
| *FLG* | 1q21 | R826X, 3222del4, R1140X, 4271delAA, Q1790X, 5757del4, 6834del5, 6950del8, S2706X and K4671X, 41delA, R501X, 3321delA, R1474X, Q2417X, E2422X, 7945delA and R4306X | Yes, FLG mutation | Chinese, Han | 261/92 | E70 |
| *FLG* | 1q21 | R501X, 2282del4 | Yes | Denmark | 3335 | E73 |
| *FLG* | 1q21 | rs7927894, R501X, 2282del4, S3247X, R2447X | Yes on others, no on rs877776 | Irish | 511/1000 children | E77 |
| *FLG* | 1q21 | R501X, 2282del4, S3247X, R2447X | Yes | Austrian, German | 462/402 | E79 |
| *FLG* | 1q21 | E2422X, Q2417X, S2554X, S2889X, S3296X, R4307X, 7945delA, 3321delA | Yes on 3321delA | Northern China | 160/169 | E80 |
| *FLG* | 1q21 | R501X, 2282del4, R2447X, S3247X, 3702delG, 3673delC | No, FLG null mutation | U.K | 792 Children aged 7-9 years | E82 |
| *FLG* | 1q21 | c.12069A>T (p.Lys4021X) | Yes, AE | Japanese | 137/134 | E83 |
| *FLG* | 1q21 | 32 SNPs covering EDC | Yes | German | 402/325 children and adults | E86 |
| *FLG* | 1q21 | R501X-, 2282del4 | Yes | Caucasian | 496 children and 488 parents families | E88 |
| *FLG2* | 1q21 | rs12568784, rs16833974 | Yes | African American | 380 AD | E30 |
| *FLT4* |  | rs10085109, rs3736062, rs11949194 | Yes on rs10085109 | Korean | 646/474, 440/692 | E40 |
| *GM-CSF* | 5q31–33 | -677A/C | Yes | Polish | 237/183 | E32 |
| *GSTM1* |  | present/null polymorphism | Yes on present polymorphism | Korean | 194/244 children | E10 |
| *GSTM1* |  | null genotype | No | korean | 124/260 preschool age children | E81 |
| *GSTP1* | 11q13 | Val105 | Yes | korean | 124/260 preschool age children | E81 |
| *GSTT1* | 22q11.2 | present/null polymorphism | No | Korean | 194/244 children | E10 |
| *GSTT1* | 22q11.2 | null genotype | No | korean | 124/260 preschool age children | E81 |
| *HNMT* |  | -465T>C, -413C>T, 314C>T, 939A>G | Yes | korean | 396 AE/124 Non-AE/146/97 | E53 |
| *HRH4* |  |  | Yes on copy numbers | Chinese | 541/613 | E39 |
| *HRH4* |  | ss142022671, ss142022677, ss142022679 | Yes | Chinese | 301/313 | E76 |
| *HRNR* | 1q21 | rs11204937, rs877776 | No | Polish | 152/104 | E8 |
| *HRNR* | 1q21 | rs7550106 | Yes | German | 939/975; replicated in 268 nuclear families | E51 |
| *IFN-γ* |  | +874T/A | Yes | Egyptian | 75/25 | E84 |
| *IFNGR1* |  | rs10457655, rs7749390 | Yes | USA | 112/166 | E61 |
| *IL-1α* |  | -889C>T | No | Croatian | 356 AD | E2 |
| *IL-1α* |  | -889 | No | Iranian | 89/140 | E44 |
| *IL-1**β* |  | -511 | No | Iranian | 89/140 | E44 |
| *IL-1β* |  | +3962 | No | Iranian | 89/140 | E44 |
| *IL-1R* |  | Pst-1 1970 | Yes | Iranian | 89/140 | E44 |
| *IL-1RA* |  | Mspa-1 11100 | No | Iranian | 89/140 | E44 |
| *IL-4* |  | C-590T | No | Egypt/Saudi Arabia | 50 non-AD/50 AD/50 children | E5 |
| *IL-4* |  | rs2243250, rs2227284 | No | Poland | 177/194 | E11 |
| *IL-4* |  | -1098, -590, -33 | Yes | Iranian | 89/139 | E12 |
| *IL-4* | 5q31.1 | -509C/T | No | Poland | 76/60 | E15 |
| *IL-4* |  | -590 C/T, -33 C/T | No | Egyptian | 106/95/100 children | E27 |
| *IL-4* |  | -1098G/T | Yes | Czech | 94/103 | E50 |
| *IL-4* |  | -590C/T | Yes | Czech | 94/103 | E50 |
| *IL-4* |  | 5kb up-stream and 5kb down-stream SNPs | No | Korean | 631/458 | E56 |
| *IL-4* |  | 590C⁄T | Yes | Eastern European/Polish descent | 163/204 | E63 |
| *IL-4R* |  | 5kb up-stream and 5kb down-stream SNPs | No | Korean | 631/458 | E56 |
| *IL-4Rα* |  | I50V | Yes | Egypt/Saudi Arabia | 50 non-AD/50 AD/50 children | E5 |
| *IL-4Rα* |  | rs1805010, rs1805011 | Yes on rs1805011 | Poland | 177/194 | E11 |
| *IL-4Rα* |  | +1902 | No | Iranian | 89/139 | E12 |
| *IL-4Rα* | 5p13 | I50V, Q576R | Yes | Egyptian | 106/95/100 children | E27 |
| *IL-4Rα* |  | rs1805011, rs1805015, rs1801275 | No, eczema | Japanese women | 188/635 | E41 |
| *IL-4Rα* |  | C-3223T, T-1914C, T-890C, Ile50Val, Glu375Ala | Yes | Japanese | 45/125 | E55 |
| *IL-6* |  | -174 | Yes | Iranian | 89/139 | E36 |
| *IL-6* |  | -174, 565A/G | Yes | Czech | 94/103 | E50 |
| *IL-6R* |  | Asp358Ala, rs2228145 | Yes with persistant AD | European, German, UK, Czech, Polish | 7130/9253 | E37 |
| *IL-7R* | 5P13 | rs12516866, rs10213865, rs1389832, rs1053496, rs10058453 | No with AD; Yes with ADEH except rs1053496 | European American | 444 subjects | E72 |
| *IL-7R* | 5P13 | rs12516866, rs10213865, rs1389832, rs1053496, rs10058453 | Yes on rs1053496 | African American | 339 subjects | E72 |
| *IL-9* |  | rs31563 (-4091G/A) | Yes | Korean | 631/459 | E65 |
| *IL-9R* |  | rs3093467 | Yes | Korean | 631/459 | E65 |
| *IL-10* | 1q31-32 | -1082G>A | No | Croatian | 356 AD | E2 |
| *IL-10* | 1q31-32 | rs1800896 (-1082G/A), rs1800871 (-819C/T), rs1800872 (-592C/A), rs1800890 (-3575T/A) | Yes | Finland | 135 preschool children | E3 |
| *IL-10* | 1q31-32 | -1082G/A | AD with this genotype associated with high serum IL-10 | Poland | 76/60 | E15 |
| *IL-10* | 1q31-32 | rs1800896 (-1082A>G) | No | UK, Taiwan, Czech, Poland, Germany, Korea, Macedonia | 849/1195 | E34 |
| *IL-10* | 1q31-32 | -1082A/G | Yes | Czech | 94/103 | E50 |
| *IL-10* | 1q31-32 | -819C/T | Yes | Czech | 94/103 | E50 |
| *IL-10* | 1q31-32 | -592A/C | Yes | Czech | 94/103 | E50 |
| *IL-10* | 1q31-32 | -1082A/G | Yes | Eastern European/Polish descent | 163/204 | E63 |
| *IL-10* | 1q31-32 | -3575, -2849, -2779, -2763,-1082, -851, -819, -592 | Yes | UK | 47/40 | E87 |
| *IL-10RA* |  | S138G | Yes | Egyptian | 25/25 | E75 |
| *IL-12A* |  | rs582504, rs582054, rs2243151 | Yes | Korean | 631/458 | E78 |
| *IL-12Rβ1* |  | rs438421, rs375947, rs438421, rs1870063 | Yes | Korean | 631/458 | E78 |
| *IL-12Rβ2* |  | rs2066446 | Yes | Korean | 631/458 | E78 |
| *IL-13* |  | -1112C/T | No | Polish | 152/123 | E6 |
| *IL-13* |  | rs20541 | No | Poland | 177/194 | E11 |
| *IL-13* | 5q31 | -1055C/T | No | Poland | 76/60 | E15 |
| *IL-13* |  | rs20541 | Yes | Korean | 412 infants | E26 |
| *IL-13* |  | rs3091307, rs20541 | Yes | Korean | 631/458 | E56 |
| *IL-13* |  | -1055 C/T | Yes | Eastern European/Polish descent | 163/204 | E63 |
| *IL-13Rα1* | x | rs2265753, rs2254672 | Yes | Korean | 631/458 | E56 |
| *IL-13Rα1* |  | 1398A>G | Yes with IgE level | Egyptian | 35/35 | E60 |
| *IL-13Rα2* |  | 5kb up-stream and 5kb down-stream SNPs | No | Korean | 631/458 | E56 |
| *IL-17A* |  | -157G/A | Yes | Polish | 166/160 | E23 |
| *IL-18* | 11q22.2 | -137G/C | Yes with FLG null mutation together | Polish | 152/123 | E6 |
| *IL-18* | 11q22.3 | rs187238 -137G/C, rs360721 -140C/G | No | Egyptian | 25 AD/25 AR/25 | E49 |
| *IL-23R* |  | 1142G/A | No | Polish | 166/160 | E23 |
| *IL-31* |  | -1066, -2057, IVS2 + 12 |  | Polish | 127/96 | E16 |
| *IL-31* |  | 2057G/A, 1066G/A, IVS2 + 12A/G | Yes | European | 60/59 | E90 |
| *IL-31* |  | rs4758680, rs7977932 | Yes on rs7977932 with AE | Taiwanese | 52 AE/61 non-atopic hand dermatitis /250 | E91 |
| *KIF3A* | 5q31 | rs2897442 | Yes, AE | Italian | 359/778 | E43 |
| *KIF3A* | 5q31 | rs2897442 | Yes | Chinese | 235/200 | E89 |
| *LAMA3* | 18q11.2 | rs8083148, rs1711450 | Yes | German | 470/320 | E20 |
| *LAMB3* | 1q32.2 | rs2566, rs2009292, etc | No | German | 470/320 | E20 |
| *LAMC2* | 1q25.3 | rs483783, rs601508, rs2274980, rs11586699 | No | German | 470/320 | E20 |
| *LCE1C* | 1q21 | rs17670505 | Yes | German | 939/975; replicated in 268 nuclear families | E51 |
| *LCE3C* | 1q21 | rs499697, rs17659389 | Yes | German | 939/975; replicated in 268 nuclear families | E51 |
| *LCE3C_LCE3B-del* |  |  | No | European | 1075/1658 | E74 |
| *LELP1* | 1q21 | rs7534334 | Yes | Polish | 152/104 | E8 |
| *Mal* |  | c.303G>A (Q101Q), -103 A>G (rs1893352),c.539 C>T (S180L) (rs8177374), c.394G>A (E132K), c.428G>A (R143Q), c.570G>C (E190D) | Yes | Japanese | 310 subjects | E64 |
| *NPSR1* |  | hopo546333 | Yes with AE at two years | Helsinki suburban area | 796 children | E29 |
| *OVOL1* | 11q13 | rs479844 | No, AE | Italian | 359/778 | E43 |
| *OVOL1* | 11q13 | rs479844 | Yes | Chinese | 235/200 | E89 |
| *SPINK5* | 5q32 | A1103G (Asn368Ser), G1156A (Asp386Asn), G1258A (Glu420Lys),  G2475T (Glu825Asp) | No | Northeast China | 91/250 | E59 |
| *SPINK5* |  | rs17718511, rs17860502, rs17718737 etc | Yes | Korean | 631/459 | E69 |
| *SPINK5* |  | rs2303070, rs6892205, rs2303064, rs2303067 | Yes on rs2303070 with AE; yes on rs6892205 with non-atopic hand dermatitis | Taiwanese | 52 AE/61 non-atopic hand dermatitis /250 | E91 |
| *SPRR3* |  | rs28989168 | Yes | German | 1870/1697 | E92 |
| *STAT6* | 12q13 | 2964G/A, 2892C/T | Yes | Egyptian | 106/95/100 children | E27 |
| *STAT6* |  | G2964 | Yes | Egypt/Saudi Arabia | 50 non-AD/50 AD/50 children | E5 |
| *TGM1* | 14q12 | rs941505 etc | rs941505 yes on Swedish | Swedish, German | 1753, 533/1996 | E45 |
| *TGM3* | 20p13 | rs6106447 etc | No association, but increased expression in AD lesions | Swedish, German | 1753, 533/1996 | E45 |
| *TGM5* | 15q15 | rs11070392 etc | No, epidermal transglutaminase genes | Swedish, German | 1753, 533/1996 | E45 |
| *TLR2* | 4q32 | A-16934T | Yes | Polish | 130 adults | E68 |
| *TLR2* |  | A-16934T | Yes | German | 136/129 adults | E85 |
| *Tmem79/Matt* |  | rs6684514 | Yes | English, UK, Irish, German, Scottish | 4245/10558 | E33 |
| *TNFα* | 6p21.3 | -308G>A, -238G>A | Yes (protective role of TNFA -308G>A) | Croatian | 356 AD | E2 |
| *TNFα* | 6p21.3 | -308, -238 | Yes | Iranian | 89/137 | E42 |
| *TSLP* | 5q22 | rs10043985, rs2289276, rs1898671, rs11466749, rs2416259 | Yes on rs11466749 with AD; yes on rs1898671 and rs2416259 with ADEH | European American | 444 subjects | E72 |
| *TSLP* | 5q22 | rs10043985, rs2289276, rs1898671, rs11466749, rs2416259 | Yes on rs10043985, rs2289276 | African American | 339 subjects | E72 |
| *TSLPR* |  | rs36139698, rs36177645, rs36133495 | Yes with AD; no with ADEH | European American | 444 subjects | E72 |
| *TSLPR* |  | rs36139698, rs36177645, rs36133495 | No | African American | 339 subjects | E72 |
| *VDR* |  | rs1544410, rs2228570, rs7975232, rs731236 | Yes on rs1544410 | Turkish | 42/96 | E4 |
| *VDR* *ApaI* |  | ApaI (rs7975232), BsmI(rs1544410), FokI(rs2228570), TaqI(rs731236) | Yes, but not FokI(rs2228570) | German | 265/265 | E47 |
| *VEGFB* |  | rs11607007 | No | Korean | 646/474, 440/692 | E40 |

*ACTL9*, actin like 9; *CCL22*, C-C Motif Chemokine 22; *CD14*, cluster-of-differentiation 14; *CYP27A1*, cytochrome P450 family 27 subfamily A member 1; *DEFB1*, defensin β 1; *ELOVL5*, fatty acid elongase 5; *FADS1*, fatty acid desaturase 1; *FADS2*, fatty acid desaturase 2; *FCER1A*, fragment of IgE receptor 1 α; *FLG*, filaggrin; *FLG2*, filaggrin 2; *FLT4*, fms related tyrosine kinase 4; *GM-CSF*, granulocyte-macrophage colony-stimulating factor; *GSTM1*, glutathione S-transferase M1; *GSTP1*, Glutathione S-transferase P1; *GSTT1*, glutathione S-transferase T1; *HNMT*, histamine N-methyltransferase; *HRH4*, Histamine receptor H4; *HRNR*, hornerin; *IFN-γ*, Interferon γ; *IFNGR1*, interferon γ receptor 1; *IL-1α*, interleukin 1 α; *IL-1β*, interleukin 1 β; *IL-1R*, interleukin 1 receptor; *IL-4*, interleukin 4; *IL-4R*, interleukin 4 receptor; *IL-4Rα*, interleukin 4 receptor α; *IL-6*, interleukin 6; *IL-6R*, interleukin 6 receptor; *IL-7R*, interleukin 7 receptor; *IL-9*, interleukin 9; *IL-9R*, interleukin 9 receptor; *IL-10*, interleukin 10; *IL-10RA*, interleukin 10 receptor α; *IL-12A*, interleukin 12 α; *IL-12R*β*1*, interleukin 12 receptor β 1; *IL-12R*β*2*, interleukin 12 receptor β 2; *IL-13*, interleukin 13; *IL-13Rα1*, interleukin 13 receptor α *1*; *IL-13Rα2*, interleukin 13 receptor α *2*; *IL-17A*, interleukin 17 α; *IL-18*, interleukin 18; *IL-23R*, interleukin 23 receptor; *IL-31*, interleukin 31; *KIF3A*, kinesin family member 3A; *LAMA3*, laminin subunit α 3; *LAMB3*, laminin subunit β 3; *LAMC2*, laminin subunit γ 2; *LCE1C*, late cornified envelope 1C; *LCE3B*, late cornified envelope 3B; *LCE3C*, late cornified envelope 3C; *LCE3C_LCE3B-del*, late cornified envelope 3C and 3B deletion; *LELP1*, latecornified envelope-like proline-rich 1; *Mal*, MyD88 adaptor-like; *NPSR1*, Neuropeptide S Receptor; *OVOL1*, ovo like transcriptional repressor 1; *SPINK5*, serine protease inhibitor Kazal type 5; *SPRR3*, small proline rich protein 3; *STAT6*, signal transducer and activator of transcription 6; *TGM1*, transglutaminase 1; *TGM3*, transglutaminase 3; *TGM5*, transglutaminase 5; *TLR2*, toll like receptor 2; *Tmem79/**Matt*, transmembrane protein 79/Mattrin; *TNF**α*, Tumor necrosis factor α; *TSLP*, thymic stromal lymphopoietin; *TSLPR*, thymic stromal lymphopoietin receptor; *VDR,* Vitamin D Receptor; *VEGFB*, vascular endothelial growth factor B.
