# Supplementary material for: Genetic and epigenetic studies of atopic dermatitis
Source: Allergy Asthma Clin Immunol. 2016 Oct 19;12:52. doi: 10.1186/s13223-016-0158-5 (PMC5069938; doi:10.1186/s13223-016-0158-5)
Supplement: Supplementary file 2 — Additional file 2: References. References of candidate gene association studies in Table S1. [file 13223_2016_158_MOESM2_ESM.docx]

**Supplemental references and Table E1**

E1: Suzuki H, Makino Y, Nagata M, Furuta J, Enomoto H, Hirota T, Tamari M, Noguchi

E. A rare variant in CYP27A1 and its association with atopic dermatitis with high

serum total IgE. Allergy. 2016 Jun 4. doi: 10.1111/all.12950. [Epub ahead of

print] PubMed PMID: 27259383.

E2: Babić Ž, SabolićPipinić I, Varnai VM, Kežić S, Macan J. Associations of TNFα

-308G>A, TNFα -238G>A, IL-1α -889C>T and IL-10 -1082G>A Genetic Polymorphisms

with Atopic Diseases: Asthma, Rhinitis and Dermatitis. Int Arch Allergy Immunol.

2016;169(4):231-40. doi: 10.1159/000445434. Epub 2016 May 31. PubMed PMID:

27240833.

E3: Korppi M, Nuolivirta K, Lauhkonen E, Holster A, Teräsjärvi J, Vuononvirta J,

Helminen M, He Q, Koponen P. IL-10 gene polymorphism is associated with preschool

atopy and early-life recurrent wheezing after bronchiolitis in infancy. Pediatr

Pulmonol. 2016 May 26. doi: 10.1002/ppul.23489. [Epub ahead of print] PubMed

PMID: 27228545.

E4: Kılıç S, Sılan F, Hız MM, Işık S, Ögretmen Z, Özdemir Ö. Vitamin D Receptor

Gene BSMI, FOKI, APAI, and TAQI Polymorphisms and the Risk of Atopic Dermatitis.

J InvestigAllergolClinImmunol. 2016;26(2):106-10. doi: 10.18176/jiaci.0020.

PubMed PMID: 27164626.

E5: Hussein YM, Alzahrani SS, Alharthi AA, Alhazmi AS, Ghonaim MM, Alghamdy AA, El

Askary A. Gene Polymorphism of Interleukin-4, Interleukin-4 Receptor and STAT6 in

Children with Atopic Dermatitis in Taif, Saudi Arabia. Immunol Invest. 2016

Apr;45(3):223-34. doi: 10.3109/08820139.2015.1135943. Epub 2016 Mar 28. PubMed

PMID: 27018548.

E6: Trzeciak M, Gleń J, Rębała K, Bandurski T, Sikorska M, Nowicki R. Coexistence

of 2282del4 FLG gene mutation and IL-18 -137G/C gene polymorphism enhances the

risk of atopic dermatitis. PostepyDermatolAlergol. 2016 Feb;33(1):57-62. doi:

10.5114/pdia.2015.48050. Epub 2016 Feb 29. PubMed PMID: 26985181; PubMed Central

PMCID: PMC4793056.

E7: Filipowska-Grońska A, Weryńska-Kalemba M, Bożek A, Filipowska B, Żebracka-Gala

J, Rusinek D, Kula D, Jarząb J. The frequency of polymorphic variants of

filaggrin gene and clinical atopic dermatitis. PostepyDermatolAlergol. 2016

Feb;33(1):37-41. doi: 10.5114/pdia.2015.48036. Epub 2016 Feb 29. PubMed PMID:

26985177; PubMed Central PMCID: PMC4793051.

E8: Trzeciak M, Wesserling M, Bandurski T, Glen J, Nowicki R, Pawelczyk T.

Association of a Single Nucleotide Polymorphism in a Late Cornified Envelope-like

Proline-rich 1 Gene (LELP1) with Atopic Dermatitis. ActaDermVenereol. 2016 Apr

12;96(4):459-63. doi: 10.2340/00015555-2301. PubMed PMID: 26608070.

E9: Bager P, Wohlfahrt J, Thyssen JP, Melbye M. Filaggrin genotype and skin

diseases independent of atopic dermatitis in childhood. Pediatr Allergy Immunol.

2016 Mar;27(2):162-8. doi: 10.1111/pai.12511. Epub 2015 Dec 21. PubMed PMID:

26594040.

E10: Chung J, Kwon SO, Ahn H, Hwang H, Hong SJ, Oh SY. Association between Dietary

Patterns and Atopic Dermatitis in Relation to GSTM1 and GSTT1 Polymorphisms in

Young Children. Nutrients. 2015 Nov 13;7(11):9440-52. doi: 10.3390/nu7115473.

PubMed PMID: 26580648; PubMed Central PMCID: PMC4663601.

E11: Narożna B, Hoffmann A, Sobkowiak P, Schoneich N, Bręborowicz A,

Szczepankiewicz A. Polymorphisms in the interleukin 4, interleukin 4 receptor and

interleukin 13 genes and allergic phenotype: A case control study. Adv Med Sci.

2016 Mar;61(1):40-5. doi: 10.1016/j.advms.2015.07.003. Epub 2015 Aug 8. PubMed

PMID: 26426602.

E12: Gharagozlou M, Behniafard N, Amirzargar AA, Hosseinverdi S, Sotoudeh S,

Farhadi E, Khaledi M, Aryan Z, Moghaddam ZG, Mahmoudi M, Aghamohammadi A, Rezaei

N. Association between single nucleotide polymorphisms of the interleukin-4 gene

and atopic dermatitis. ActaDermatovenerol Croat. 2015;23(2):96-100. PubMed PMID:

26228820.

E13: Esparza-Gordillo J, Matanovic A, Marenholz I, Bauerfeind A, Rohde K, Nemat K,

Lee-Kirsch MA, Nordenskjöld M, Winge MC, Keil T, Krüger R, Lau S, Beyer K, Kalb

B, Niggemann B, Hübner N, Cordell HJ, Bradley M, Lee YA. Maternal filaggrin

mutations increase the risk of atopic dermatitis in children: an effect

independent of mutation inheritance. PLoS Genet. 2015 Mar 10;11(3):e1005076. doi:

10.1371/journal.pgen.1005076. eCollection 2015 Mar. PubMed PMID: 25757221; PubMed

Central PMCID: PMC4355615.

E14: Thyssen JP, Tang L, Husemoen LL, Stender S, Szecsi PB, Menné T, Johansen JD,

Linneberg A. Filaggrin gene mutations are not associated with food and

aeroallergen sensitization without concomitant atopic dermatitis in adults. J

Allergy ClinImmunol. 2015 May;135(5):1375-8.e1.

E15: Lesiak A, Zakrzewski M, Przybyłowska K, Rogowski-Tylman M, Wozniacka A,

Narbutt J. Atopic dermatitis patients carrying G allele in -1082 G/A IL-10

polymorphism are predisposed to higher serum concentration of IL-10. Arch Med

Sci. 2014 Dec 22;10(6):1239-43.

E16: Sokołowska-Wojdyło M, Gleń J, Zabłotna M, Rębała K, Trzeciak M, Sikorska M,

Ruckemann-Dziurdzińska K, Nedoszytko B, Florek A, Nowicki R. The frequencies of

haplotypes defined by three polymorphisms of the IL-31 gene: -1066, -2057, and

IVS2+12 in Polish patients with atopic dermatitis. Int J Dermatol. 2015

Jan;54(1):62-7.

E17: Wang IJ, Lin TJ. FLG P478S polymorphisms and environmental risk factors for

the atopic march in Taiwanese children: a prospective cohort study. Ann Allergy

Asthma Immunol. 2015 Jan;114(1):52-7.

E18: Wang IJ, Karmaus WJ. The effect of phthalate exposure and filaggrin gene

variants on atopic dermatitis. Environ Res. 2015 Jan;136:213-8.

E19: Komova EG, Shintyapina AB, Makarova SI, Ivanov MK, Chekryga EA, Kaznacheeva

LF, Vavilin VA. Filaggrin mutations in a Western siberian population and their

association with atopic dermatitis in children. Genet Test Mol Biomarkers. 2014

Dec;18(12):791-6.

E20: Stemmler S, Parwez Q, Petrasch-Parwez E, Epplen JT, Hoffjan S. Association of

variation in the LAMA3 gene, encoding the alpha-chain of laminin 5, with atopic

dermatitis in a German case-control cohort. BMC Dermatol. 2014 Nov 3;14:17. doi:

10.1186/1471-5945-14-17. PubMed PMID: 25363238; PubMed Central PMCID: PMC4221780.

E21: Rupnik H, Rijavec M, Korošec P. Filaggrin loss-of-function mutations are not

associated with atopic dermatitis that develops in late childhood or adulthood.

Br J Dermatol. 2015 Feb;172(2):455-61.

E22: Khaledi M, Fotouhi A, Farhadi E, Mahdaviani B, Sotoudeh S, Tavakoli M,

Behniafard N, Gharagozlou M, Rezaei N. Filaggrin single nucleotide polymorphisms

in atopic dermatitis. ActaDermatovenerol Croat. 2014;22(3):200-4.

E23: Narbutt J, Wojtczak M, Zalińska A, Salinski A, Przybylowska-Sygut K, Kuna P,

Majak P, Sysa-Jedrzejowska A, Lesiak A. The A/A genotype of an interleukin-17A

polymorphism predisposes to increased severity of atopic dermatitis and

coexistence with asthma. ClinExpDermatol. 2015 Jan;40(1):11-6.

E24: Sasaki T, Furusyo N, Shiohama A, Takeuchi S, Nakahara T, Uchi H, Hirota T,

Tamari M, Shimizu N, Ebihara T, Amagai M, Furue M, Hayashi J, Kudoh J. Filaggrin

loss-of-function mutations are not a predisposing factor for atopic dermatitis in

anIshigaki Island under subtropical climate. J Dermatol Sci. 2014

Oct;76(1):10-5.

E25: Meng L, Wang L, Tang H, Tang X, Jiang X, Zhao J, Gao J, Li B, Fu X, Chen Y,

Yao W, Zhan W, Wu B, Duan D, Shen C, Cheng H, Zuo X, Yang S, Sun L, Zhang X.

Filaggrin gene mutation c.3321delA is associated with various clinical features

of atopic dermatitis in the Chinese Han population. PLoS One. 2014 May

23;9(5):e98235. doi: 10.1371/journal.pone.0098235. eCollection 2014. PubMed PMID:

24858702; PubMed Central PMCID: PMC4032331.

E26: Lee SY, Yu J, Ahn KM, Kim KW, Shin YH, Lee KS, Hong SA, Jung YH, Lee E, Yang

SI, Seo JH, Kwon JW, Kim BJ, Kim HB, Kim WK, Song DJ, Jang GC, Shim JY, Lee SY,

Kwon JY, Choi SJ, Lee KJ, Park HJ, Won HS, Yoo HS, Kang MJ, Kim HY, Hong SJ.

Additive effect between IL-13 polymorphism and cesarean section delivery/prenatal

antibiotics use on atopic dermatitis: a birth cohort study (COCOA). PLoS One.

2014 May 21;9(5):e96603. doi: 10.1371/journal.pone.0096603. eCollection 2014.

PubMed PMID: 24848505; PubMed Central PMCID: PMC4029558.

E27: Hussein YM, Shalaby SM, Nassar A, Alzahrani SS, Alharbi AS, Nouh M.

Association between genes encoding components of the IL-4/IL-4 receptor pathway

and dermatitis in children. Gene. 2014 Jul 25;545(2):276-81.

E28: Kim SY, Yang SW, Kim HL, Kim SH, Kim SJ, Park SM, Son M, Ryu S, Pyo YS, Lee

JS, Kim KS, Kim YB, Hong SH, Um JY. Association between P478S polymorphism of the

filaggrin gene & atopic dermatitis. Indian J Med Res. 2013 Dec;138(6):922-7.

E29: P K, M K, K K, T L, M K. Interaction of NPSR1 genotypes and probiotics in the

manifestation of atopic eczema in early childhood. AllergolImmunopathol (Madr).

2014 Nov-Dec;42(6):560-7.

E30: Margolis DJ, Gupta J, Apter AJ, Ganguly T, Hoffstad O, Papadopoulos M,

Rebbeck TR, Mitra N. Filaggrin-2 variation is associated with more persistent

atopic dermatitis in African American subjects. J Allergy ClinImmunol. 2014

Mar;133(3):784-9.

E31: Chisaguano AM, Montes R, Pérez-Berezo T, Castellote AI, Guerendiain M,

Bustamante M, Morales E, García-Esteban R, Sunyer J, Franch A, López-Sabater MC.

Gene expression of desaturase (FADS1 and FADS2) and Elongase (ELOVL5) enzymes in

peripheral blood: association with polyunsaturated fatty acid levels and atopic

eczema in 4-year-old children. PLoS One. 2013 Oct 22;8(10):e78245. doi:

10.1371/journal.pone.0078245. eCollection 2013. PubMed PMID: 24167612; PubMed

Central PMCID: PMC3805510.

E32: Wilkowska A, Gleń J, Zabłotna M, Trzeciak M, Ryduchowska M, Sobjanek M,

Nedoszytko B, Nowicki R, Sokołowska-Wojdyło M. The association of GM-CSF -677A/C

promoter gene polymorphism with the occurrence and severity of atopic dermatitis

in a Polish population. Int J Dermatol. 2014 Mar;53(3):e172-4. doi:

10.1111/ijd.12245. Epub 2013 Oct 14. PubMed PMID: 24117406.

E33: Saunders SP, Goh CS, Brown SJ, Palmer CN, Porter RM, Cole C, Campbell LE,

Gierlinski M, Barton GJ, Schneider G, Balmain A, Prescott AR, Weidinger S,

Baurecht H, Kabesch M, Gieger C, Lee YA, Tavendale R, Mukhopadhyay S, Turner SW,

Madhok VB, Sullivan FM, Relton C, Burn J, Meggitt S, Smith CH, Allen MA, Barker

JN, Reynolds NJ, Cordell HJ, Irvine AD, McLean WH, Sandilands A, Fallon PG.

Tmem79/Matt is the matted mouse gene and is a predisposing gene for atopic

dermatitis in human subjects. J Allergy ClinImmunol. 2013 Nov;132(5):1121-9.

E34: Chen S, Zhao L. Association between the IL10 -1082A>G (rs1800896)

single-nucleotide polymorphism and atopic dermatitis: a systematic review and

meta-analysis. Dermatitis. 2013 Jul-Aug;24(4):161-5

E35: Yu HS, Kang MJ, Jung YH, Kim HY, Seo JH, Kim YJ, Lee SH, Kim HJ, Kwon JW, Kim

BJ, Yu J, Hong SJ. Mutations in the Filaggrin are Predisposing Factor in Korean

Children With Atopic Dermatitis. Allergy Asthma Immunol Res. 2013 Jul;5(4):211-5.

E36: Gharagozlou M, Farhadi E, Khaledi M, Behniafard N, Sotoudeh S, Salari R,

Darabi B, Fathi SM, Mahmoudi M, Aghamohammadi A, Amirzargar AA, Rezaei N.

Association between the interleukin 6 genotype at position -174 and atopic

dermatitis. J InvestigAllergolClinImmunol. 2013;23(2):89-93.

E37: Esparza-Gordillo J, Schaarschmidt H, Liang L, Cookson W, Bauerfeind A,

Lee-Kirsch MA, Nemat K, Henderson J, Paternoster L, Harper JI, Mangold E, Nothen

MM, Rüschendorf F, Kerscher T, Marenholz I, Matanovic A, Lau S, Keil T, Bauer CP,

Kurek M, Ciechanowicz A, Macek M, Franke A, Kabesch M, Hubner N, Abecasis G,

Weidinger S, Moffatt M, Lee YA. A functional IL-6 receptor (IL6R) variant is a

risk factor for persistent atopic dermatitis. J Allergy ClinImmunol. 2013

Aug;132(2):371-7.

E38: Linneberg A, Fenger RV, Husemoen LL, Thuesen BH, Skaaby T, Gonzalez-Quintela

A, Vidal C, Carlsen BC, Johansen JD, Menné T, Stender S, Melgaard M, Szecsi PB,

Berg ND, Thyssen JP. Association between loss-of-function mutations in the

filaggrin gene and self-reported food allergy and alcohol sensitivity. Int Arch

Allergy Immunol. 2013;161(3):234-42.

E39: Chen B, Ye T, Shao Y, Zhang J, Zhong Q, Hu X, Zhang W, Yu B. Association

between copy-number variations of the human histamine H4 receptor gene and atopic

dermatitis in a Chinese population. ClinExpDermatol. 2013 Apr;38(3):295-300;

E40: Namkung JH, Lee JE, Kim E, Huh IS, Park T, Shin ES, Cho EY, Yang JM. Single

nucleotide polymorphism in the FLT4 gene is associated with atopic dermatitis in

Koreans. Cytokine. 2013 Apr;62(1):110-4.

E41: Miyake Y, Tanaka K, Arakawa M. Case-control study of eczema in relation to

IL4Rα genetic polymorphisms in Japanese women: The Kyushu Okinawa Maternal and

Child Health Study. Scand J Immunol. 2013 May;77(5):413-8.

E42: Behniafard N, Gharagozlou M, Farhadi E, Khaledi M, Sotoudeh S, Darabi B,

Fathi SM, GholizadehMoghaddam Z, Mahmoudi M, Aghamohammadi A, Amirzargar AA,

Rezaei N. TNF-alpha single nucleotide polymorphisms in atopic dermatitis. Eur

Cytokine Netw. 2012 Oct-Dec;23(4):163-5.

E43: Lepre T, Cascella R, Ragazzo M, Galli E, Novelli G, Giardina E. Association

of KIF3A, but not OVOL1 and ACTL9, with atopic eczema in Italian patients. Br J

Dermatol. 2013 May;168(5):1106-8.

E44: Behniafard N, Gharagozlou M, Sotoudeh S, Farhadi E, Khaledi M, Moghaddam ZG,

Mahmoudi M, Fathi SM, Darabi B, Aghamohammadi A, Amirzargar AA, Rezaei N.

Association of single nucleotide polymorphisms of interleukin-1 family with

atopic dermatitis. AllergolImmunopathol (Madr). 2014 May-Jun;42(3):212-5.

E45: Liedén A, Winge MC, Sääf A, Kockum I, Ekelund E, Rodriguez E, Fölster-Holst

R, Franke A, Illig T, Tengvall-Linder M, Baurecht H, Weidinger S, Wahlgren CF,

Nordenskjöld M, Bradley M. Genetic variation in the epidermal transglutaminase

genes is not associated with atopic dermatitis. PLoS One. 2012;7(11):e49694. doi:

10.1371/journal.pone.0049694.

E46: Cheng R, Li M, Zhang H, Guo Y, Chen X, Tao J, Jiang A, Gan J, Qi H, Yu H,

Liao W, Yao Z. Common FLG mutation K4671X not associated with atopic dermatitis

in Han Chinese in a family association study. PLoS One. 2012;7(11):e49158. doi:

10.1371/journal.pone.0049158.

E47: Heine G, Hoefer N, Franke A, Nöthling U, Schumann RR, Hamann L, Worm M.

Association of vitamin D receptor gene polymorphisms with severe atopic

dermatitis in adults. Br J Dermatol. 2013 Apr;168(4):855-8.

E48: Li M, Liu JB, Liu Q, Yao M, Cheng R, Xue H, Zhou H, Yao Z. Interactions

between FLG mutations and allergens in atopic dermatitis. Arch Dermatol Res. 2012

Dec;304(10):787-93.

E49: Ibrahim GH, ElTabbakh MT, Gomaa AH, Mohamed EA. Interleukin-18 gene

polymorphisms in Egyptian patients with allergic diseases. Am J Rhinol Allergy.

2012 Sep-Oct;26(5):385-9.

E50: Kayserova J, Sismova K, Zentsova-Jaresova I, Katina S, Vernerova E,

Polouckova A, Capkova S, Malinova V, Striz I, Sediva A. A prospective study in

children with a severe form of atopic dermatitis: clinical outcome in relation to

cytokine gene polymorphisms. J InvestigAllergolClinImmunol. 2012;22(2):92-101.

E51: Knüppel S, Esparza-Gordillo J, Marenholz I, Holzhütter HG, Bauerfeind A,

Ruether A, Weidinger S, Lee YA, Rohde K. Multi-locus stepwise regression: a

haplotype-based algorithm for finding genetic associations applied to atopic

dermatitis. BMC Med Genet. 2012 Jan 27;13:8. doi: 10.1186/1471-2350-13-8.

E52: Zhou J, Zhou Y, Lin LH, Wang J, Peng X, Li J, Li L. Association of

polymorphisms in the promoter region of FCER1A gene with atopic dermatitis,

chronicuticaria, asthma, and serum immunoglobulin E levels in a Han Chinese

population. Hum Immunol. 2012 Mar;73(3):301-5.

E53: Lee HS, Kim SH, Kim KW, Baek JY, Park HS, Lee KE, Hong JY, Kim MN, Heo WI,

Sohn MH, Kim KE. Involvement of human histamine N-methyltransferase gene

polymorphisms in susceptibility to atopic dermatitis in korean children. Allergy

Asthma Immunol Res. 2012 Jan;4(1):31-6. doi: 10.4168/aair.2012.4.1.31. Epub 2011

Nov 10. Erratum in: Allergy Asthma Immunol Res. 2013 Mar;5(2):116. PubMed PMID:

22211168; PubMed Central PMCID: PMC3242058.

E54: Hirota T, Saeki H, Tomita K, Tanaka S, Ebe K, Sakashita M, Yamada T, Fujieda

S, Miyatake A, Doi S, Enomoto T, Hizawa N, Sakamoto T, Masuko H, Sasaki T,

Ebihara T, Amagai M, Esaki H, Takeuchi S, Furue M, Noguchi E, Kamatani N,

Nakamura Y, Kubo M, Tamari M. Variants of C-C motif chemokine 22 (CCL22) are

associated with susceptibility to atopic dermatitis: case-control studies. PLoS

One. 2011;6(11):e26987. doi: 10.1371/journal.pone.0026987.

E55: Tanaka T, Hitomi Y, Kambayashi Y, Hibino Y, Fukutomi Y, Shibata A, Sugimoto

N, Hatta K, Eboshida A, Konoshita T, Nakamura H. The differences in the

involvements of loci of promoter region and Ile50Val in interleukin-4 receptor α

chain gene between atopic dermatitis and Japanese cedar pollinosis. Allergol Int.

2012 Mar;61(1):57-63.

E56: Namkung JH, Lee JE, Kim E, Kim HJ, Seo EY, Jang HY, Shin ES, Cho EY, Yang JM.

Association of polymorphisms in genes encoding IL-4, IL-13 and their receptors

with atopic dermatitis in a Korean population. ExpDermatol. 2011

Nov;20(11):915-9.

E57: Cai SC, Chen H, Koh WP, Common JE, van Bever HP, McLean WH, Lane EB, Giam YC,

Tang MB. Filaggrin mutations are associated with recurrent skin infection in

Singaporean Chinese patients with atopic dermatitis. Br J Dermatol. 2012

Jan;166(1):200-3.

E58: Park KY, Park MK, Kim EJ, Lee MK, Seo SJ. FCεRI gene promoter polymorphisms

and total IgE levels in susceptibility to atopic dermatitis in Korea. J Korean

Med Sci. 2011 Jul;26(7):870-4.

E59: Zhao LP, Di Z, Zhang L, Wang L, Ma L, Lv Y, Hong Y, Wei H, Chen HD, Gao XH.

Association of SPINK5 gene polymorphisms with atopic dermatitis in Northeast

China. J EurAcadDermatolVenereol. 2012 May;26(5):572-7.

E60: Hussein YM, Ahmad AS, Ibrahem MM, Elsherbeny HM, Shalaby SM, El-Shal AS,

Sabbah NA. Interleukin 13 receptors as biochemical markers in atopic patients. J

InvestigAllergolClinImmunol. 2011;21(2):101-7.

E61: Leung DY, Gao PS, Grigoryev DN, Rafaels NM, Streib JE, Howell MD, Taylor PA,

Boguniewicz M, Canniff J, Armstrong B, Zaccaro DJ, Schneider LC, Hata TR, Hanifin

JM, Beck LA, Weinberg A, Barnes KC. Human atopic dermatitis complicated by eczema

herpeticum is associated with abnormalities in IFN-γ response. J Allergy Clin

Immunol. 2011 Apr;127(4):965-73.e1-5. doi: 10.1016/j.jaci.2011.02.010. Erratum

in: J Allergy ClinImmunol. 2011 Oct;128(4):833. PubMed PMID: 21458658; PubMed

Central PMCID: PMC3074534.

E62: Chen H, Common JE, Haines RL, Balakrishnan A, Brown SJ, Goh CS, Cordell HJ,

Sandilands A, Campbell LE, Kroboth K, Irvine AD, Goh DL, Tang MB, van Bever HP,

Giam YC, McLean WH, Lane EB. Wide spectrum of filaggrin-null mutations in atopic

dermatitis highlights differences between Singaporean Chinese and European

populations. Br J Dermatol. 2011 Jul;165(1):106-14.

E63: Lesiak A, Kuna P, Zakrzewski M, van Geel M, Bladergroen RS, Przybylowska K,

Stelmach I, Majak P, Hawro T, Sysa-Jedrzejowska A, Narbutt J. Combined occurrence

offilaggrin mutations and IL-10 or IL-13 polymorphisms predisposes to atopic

dermatitis. ExpDermatol. 2011 Jun;20(6):491-5.

E64: An Y, Ohnishi H, Matsui E, Funato M, Kato Z, Teramoto T, Kaneko H, Kimura T,

Kubota K, Kasahara K, Kondo N. Genetic variations in MyD88 adaptor-like are

associated with atopic dermatitis. Int J Mol Med. 2011 Jun;27(6):795-801.

E65: Namkung JH, Lee JE, Kim E, Park GT, Yang HS, Jang HY, Shin ES, Cho EY, Yang

JM. An association between IL-9 and IL-9 receptor gene polymorphisms and atopic

dermatitis in a Korean population. J Dermatol Sci. 2011 Apr;62(1):16-21.

E66: Cascella R, FotiCuzzola V, Lepre T, Galli E, Moschese V, Chini L, Mazzanti

C, Fortugno P, Novelli G, Giardina E. Full sequencing of the FLG gene in Italian

patients with atopic eczema: evidence of new mutations, but lack of an

association. J Invest Dermatol. 2011 Apr;131(4):982-4.

E67: Wang IJ, Lin TJ, Kuo CF, Lin SL, Lee YL, Chen PC. Filaggrin polymorphism

P478S, IgE level, and atopic phenotypes. Br J Dermatol. 2011 Apr;164(4):791-6.

E68: Potaczek DP, Nastalek M, Okumura K, Wojas-Pelc A, Undas A, Nishiyama C. An

association of TLR2–16934A >T polymorphism and severity/phenotype of atopic

dermatitis. J EurAcadDermatolVenereol. 2011 Jun;25(6):715-21.

E69: Namkung JH, Lee JE, Kim E, Byun JY, Kim S, Shin ES, Cho EY, Yang JM. Hint for

association of single nucleotide polymorphisms and haplotype in SPINK5 gene with

atopic dermatitis in Koreans. ExpDermatol. 2010 Dec;19(12):1048-53.

E70: Zhang H, Guo Y, Wang W, Shi M, Chen X, Yao Z. Mutations in the filaggrin gene

in Han Chinese patients with atopic dermatitis. Allergy. 2011 Mar;66(3):420-7.

E71: Segat L, Guimarães RL, Brandão LA, Rocha CR, Zanin V, Trevisiol C, de Lima

Filho JL, Crovella S. Beta defensin-1 gene (DEFB1) polymorphisms are not

associated with atopic dermatitis in children and adolescents from northeast

Brazil (Recife, Pernambuco). Int J Dermatol. 2010 Jun;49(6):653-7.

E72: Gao PS, Rafaels NM, Mu D, Hand T, Murray T, Boguniewicz M, Hata T, Schneider

L, Hanifin JM, Gallo RL, Gao L, Beaty TH, Beck LA, Leung DY, Barnes KC. Genetic

variants in thymic stromal lymphopoietin are associated with atopic dermatitis

and eczema herpeticum. J Allergy ClinImmunol. 2010 Jun;125(6):1403-1407.e4.

E73: Thyssen JP, Carlsen BC, Menné T, Linneberg A, Nielsen NH, Meldgaard M, Szecsi

PB, Stender S, Johansen JD. Filaggrin null mutations increase the risk and

persistence of hand eczema in subjects with atopic dermatitis: results from a

general population study. Br J Dermatol. 2010 Jul;163(1):115-20.

E74: Bergboer JG, Zeeuwen PL, Irvine AD, Weidinger S, Giardina E, Novelli G, Den

Heijer M, Rodriguez E, Illig T, Riveira-Munoz E, Campbell LE, Tyson J, Dannhauser

EN, O'Regan GM, Galli E, Klopp N, Koppelman GH, Novak N, Estivill X, McLean WH,

Postma DS, Armour JA, Schalkwijk J. Deletion of Late Cornified Envelope 3B and 3C

genes is not associated with atopic dermatitis. J Invest Dermatol. 2010

Aug;130(8):2057-61.

E75: Hussein PY, Zahran F, AshourWahba A, Ahmad AS, Ibrahiem MM, Shalaby SM, El

Tarhouny SA, El Sherbiny HM, Bakr N. Interleukin 10 receptor alpha subunit

(IL-10RA) gene polymorphism and IL-10 serum levels in Egyptian atopic patients. J

InvestigAllergolClinImmunol. 2010;20(1):20-6.

E76: Yu B, Shao Y, Zhang J, Dong XL, Liu WL, Yang H, Liu L, Li MH, Yue CF, Fang

ZY, Zhang C, Hu XP, Chen BC, Wu Q, Chen YW, Zhang W, Wan J. Polymorphisms in

human histamine receptor H4 gene are associated with atopic dermatitis. Br J

Dermatol. 2010 May;162(5):1038-43.

E77: O'Regan GM, Campbell LE, Cordell HJ, Irvine AD, McLean WH, Brown SJ.

Chromosome 11q13.5 variant associated with childhood eczema: an effect

supplementary to filaggrin mutations. J Allergy ClinImmunol. 2010

Jan;125(1):170-4.e1-2.

E78: Namkung JH, Lee JE, Kim E, Kim S, Kim S, Shin ES, Cho EY, Yang JM.

Association of single nucleotide polymorphisms in the IL-12 (IL-12A and B) and

IL-12 receptor (IL-12Rbeta1 and beta2) genes and gene-gene interactions with

atopic dermatitis in Koreans. J Dermatol Sci. 2010 Mar;57(3):199-206.

E79: Greisenegger E, Novak N, Maintz L, Bieber T, Zimprich F, Haubenberger D,

Gleiss A, Stingl G, Kopp T, Zimprich A. Analysis of four prevalent filaggrin

mutations (R501X, 2282del4, R2447X and S3247X) in Austrian and German patients

with atopic dermatitis. J EurAcadDermatolVenereol. 2010 May;24(5):607-10.

E80: Ma L, Zhang L, Di ZH, Zhao LP, Lu YN, Xu J, Chen HD, Gao XH. Association

analysis of filaggrin gene mutations and atopic dermatitis in Northern China. Br

J Dermatol. 2010 Jan;162(1):225-7.

E81: Chung J, Oh SY, Shin YK. Association of glutathione-S-transferase

polymorphisms with atopic dermatitis risk in preschool age children. ClinChem

Lab Med. 2009;47(12):1475-81.

E82: Brown SJ, Relton CL, Liao H, Zhao Y, Sandilands A, McLean WH, Cordell HJ,

Reynolds NJ. Filaggrinhaploinsufficiency is highly penetrant and is associated

with increased severity of eczema: further delineation of the skin phenotype in a

prospective epidemiological study of 792 school children. Br J Dermatol. 2009

Oct;161(4):884-9.

E83: Nemoto-Hasebe I, Akiyama M, Nomura T, Sandilands A, McLean WH, Shimizu H.

FLG mutation p.Lys4021X in the C-terminal imperfect filaggrin repeat in Japanese

patients with atopic eczema. Br J Dermatol. 2009 Dec;161(6):1387-90.

E84: Hussein YM, Ahmad AS, Ibrahem MM, El Tarhouny SA, Shalaby SM, Elshal AS, El

Said M. Interferon gamma gene polymorphism as a biochemical marker in Egyptian

atopic patients. J InvestigAllergolClinImmunol. 2009;19(4):292-8.

E85: Oh DY, Schumann RR, Hamann L, Neumann K, Worm M, Heine G. Association of the

toll-like receptor 2 A-16934T promoter polymorphism with severe atopic

dermatitis. Allergy. 2009 Nov;64(11):1608-15.

E86: Stemmler S, Nothnagel M, Parwez Q, Petrasch-Parwez E, Epplen JT, Hoffjan S.

Variation in genes of the epidermal differentiation complex in German atopic

dermatitis patients. Int J Immunogenet. 2009 Aug;36(4):217-22.

E87: Lacy K, Archer C, Wood N, Bidwell J. Association between a common IL10

distal promoter haplotype and IgE production in individuals with atopic

dermatitis. Int J Immunogenet. 2009 Aug;36(4):213-6.

E88: Müller S, Marenholz I, Lee YA, Sengler C, Zitnik SE, Griffioen RW, Meglio P,

Wahn U, Nickel R. Association of Filaggrin loss-of-function-mutations with atopic

dermatitis and asthma in the Early Treatment of the Atopic Child (ETAC)

population. Pediatr Allergy Immunol. 2009 Jun;20(4):358-61.

E89: Kang Z, Li Q, Fu P, Yan S, Guan M, Xu J, Xu F. Correlation of KIF3A and OVOL1,

but not ACTL9, with atopic dermatitis in Chinese pediatric patients. Gene. 2015

Oct 25;571(2):249-51. doi: 10.1016/j.gene.2015.06.068. Epub 2015 Jun 27. PubMed

PMID: 26127003.

E90: Sokołowska-Wojdyło M, Gleń J, Zabłotna M, Rębała K, Sikorska M, Florek A,

Trzeciak M, Barańska-Rybak W, Malek M, Nedoszytko B. Association of distinct

IL-31 polymorphisms with pruritus and severity of atopic dermatitis. J EurAcad

DermatolVenereol. 2013 May;27(5):662-4. doi: 10.1111/j.1468-3083.2012.04649.x.

Epub 2012 Jul 24. PubMed PMID: 22827739.

E91: Lan CC, Tu HP, Wu CS, Ko YC, Yu HS, Lu YW, Li WC, Chen YC, Chen GS. Distinct

SPINK5 and IL-31 polymorphisms are associated with atopic eczema and non-atopic

hand dermatitis in Taiwanese nursing population. ExpDermatol. 2011

Dec;20(12):975-9. doi: 10.1111/j.1600-0625.2011.01374.x. Epub 2011 Oct 20. PubMed

PMID: 22017185.

E92: Marenholz I, [Rivera VA](http://www.ncbi.nlm.nih.gov/pubmed/?term=Rivera%20VA%5BAuthor%5D&cauthor=true&cauthor_uid=21490620), [Esparza-Gordillo J](http://www.ncbi.nlm.nih.gov/pubmed/?term=Esparza-Gordillo%20J%5BAuthor%5D&cauthor=true&cauthor_uid=21490620), [Bauerfeind A](http://www.ncbi.nlm.nih.gov/pubmed/?term=Bauerfeind%20A%5BAuthor%5D&cauthor=true&cauthor_uid=21490620), [Lee-Kirsch MA](http://www.ncbi.nlm.nih.gov/pubmed/?term=Lee-Kirsch%20MA%5BAuthor%5D&cauthor=true&cauthor_uid=21490620), [Ciechanowicz A](http://www.ncbi.nlm.nih.gov/pubmed/?term=Ciechanowicz%20A%5BAuthor%5D&cauthor=true&cauthor_uid=21490620),

[Kurek M](http://www.ncbi.nlm.nih.gov/pubmed/?term=Kurek%20M%5BAuthor%5D&cauthor=true&cauthor_uid=21490620), [Piskackova T](http://www.ncbi.nlm.nih.gov/pubmed/?term=Piskackova%20T%5BAuthor%5D&cauthor=true&cauthor_uid=21490620), [Macek M](http://www.ncbi.nlm.nih.gov/pubmed/?term=Macek%20M%5BAuthor%5D&cauthor=true&cauthor_uid=21490620), [Lee YA](http://www.ncbi.nlm.nih.gov/pubmed/?term=Lee%20YA%5BAuthor%5D&cauthor=true&cauthor_uid=21490620).Association screening in the Epidermal Differentiation

Complex (EDC) identifies an SPRR3 repeat number variant as a risk factor for eczema. The Journal of

investigative dermatology. 2011 AUG; 131(8):1644-1649.
